# Formation of friable embryogenic callus in cassava is enhanced under conditions of reduced nitrate, potassium and phosphate

**DOI:** 10.1371/journal.pone.0180736

**Published:** 2017-08-14

**Authors:** Yoshinori Utsumi, Chikako Utsumi, Maho Tanaka, Vu The Ha, Akihiro Matsui, Satoshi Takahashi, Motoaki Seki

**Affiliations:** 1 Plant Genomic Network Research Team, RIKEN Center for Sustainable Resource Science, 1-7-22 Suehiro-cho, Tsurumi-ku, Yokohama, Kanagawa, Japan; 2 Core Research for Evolutional Science and Technology (CREST), Japan Science and Technology (JST), 4-1-8 Honcho, Kawaguchi, Saitama, Japan; 3 Kihara Institute for Biological Research, Yokohama City University, 641–12 Maioka-cho, Totsuka-ku, Yokohama, Kanagawa, Japan; Shanghai Institutes for Biological Sciences, CHINA

## Abstract

*Agrobacterium*-mediated transformation is an important research tool for the genetic improvement of cassava. The induction of friable embryogenic callus (FEC) is considered as a key step in cassava transformation. In the present study, the media composition was optimized for enhancing the FEC induction, and the effect of the optimized medium on gene expression was evaluated. In relative comparison to MS medium, results demonstrated that using a medium with reducing nutrition (a 10-fold less concentration of nitrogen, potassium, and phosphate), the increased amount of vitamin B1 (10 mg/L) and the use of picrolam led to reprogram non-FEC to FEC. Gene expression analyses revealed that FEC on modified media increased the expression of genes related to the regulation of polysaccharide biosynthesis and breakdown of cell wall components in comparison to FEC on normal CIM media, whereas the gene expression associated with energy flux was not dramatically altered. It is hypothesized that we reprogram non-FEC to FEC under low nitrogen, potassium and phosphate and high vitamin B1. These findings were more effective in inducing FEC formation than the previous protocol. It might contribute to development of an efficient transformation strategy in cassava.

## Introduction

Cassava (*Manihot esculenta* Crantz) is grown throughout the tropics for its production of starchy roots. It is widely used in Africa and Asia for human consumption, animal feed, and as an industrial material [1–3]. Although cassava is an important tropical crop, conventional breeding programs, based on sexual hybridization, have not been well developed. Cassava breeding is problematic due to the high degree of genetic heterozygosity, genetic overloading, separation of progeny, few flowers, low pollen fertility, self-incompatibility, and low fruit set [4, 5]. Therefore, optimization of a genetic transformation system for the molecular breeding of cassava plants is critical to overcome the problems associated with conventional breeding approaches.

Several studies have reported the establishment of a cassava transformation system for creating transgenic cassava plants. Development of a culture system using somatic embryos was reported by Stamp and Henshaw [6]. Taylor et al. [7] developed a cassava culture system that produced friable embryogenic callus (FEC) from somatic embryos. Li et al. [8] reported the *Agrobacterium*-mediated transformation and Schöpke et al. [9] and Zhang et al. [10] reported about the particle bombardment-mediated transformation of cassava, respectively. *Agrobacterium*-mediated transformation via FEC has been used as a standard and reproducible method for transformation of cassava including model cassava cultivar 60444 [11–28], and it has been used to develop transgenic cassava plants with resistance to African cassava mosaic virus (ACMV) [29, 30], resistance to Sri LanKan cassava mosaic virus [31], resistance to cassava brown streak disease (caused by Cassava brown streak virus) [32] leaf retention and drought tolecance [33], tolerance to post-harvest physiological deterioration (PPD) by reactive oxygen species (ROS) scavengers [34–36], increased accumulation of provitamin A and B_6_ [37, 38], reduced cyanogenic glucoside content [39], increased tuber yield [40], modified starch quality (amylose-free or low amylose starch) [41, 42], and iron biofortification [43].

Although these researches using efficient transformation system have been developed mainly by the use of model cassava cultivar 60444 due to its good capacity of FEC formation and its good regeneration capacity of embryogenic tissue, 60444 has not been cultivated by local farmers in Africa due to low yield, low nutritional quality and high sensitivity to viral and bacterial disease. The transformation system using other cultivars has been reported in a few cultivars but the following problems and/or optimization of several factors still remain: 1) which tissues, culture medium and genotypes should be used; 2) difficulty of the tissue preparation; 3) low regeneration efficiency of FEC [5, 21, 22]. The farmer-preferred germplasms in West and East African has been used as materials for cassava transformation, and the process of FEC production has been achieved successfully by the addition of tyrosine, the culture under light condition, and *Agrobacterium* cell densities (OD600 nm of 0.1–0.5) at co-culture [23–28]. On the other hand, the development of healthy plants by tissue culture and synthetic seeds to farmers have been discussed as well as plant biotechnology such as transformation offers a wide range of opportunities [44]. Recently, the Next Generation Cassava Breeding (NextGen Cassava) project has been carried out in Uganda in cooperation with various institutes and universities (http://www.nextgencassava.org/). The NextGen Cassava project aims to develop the human and infrastructure capacity at partner breeding programs and advance the full potential of cassava towards contribution to food security and livelihoods by breeding method knowns as genomic selection.

In the present study, an attempt was made to improve the protocol used to induce the generation of FEC, a prerequisite for *Agrobacterium*-mediated transformation of cassava. After the induction of somatic embryos, the cultivation of cassava tissue on Gresshoff & Doy (GD) medium has been reported as the most effective method for inducing the formation of FEC. The use of nitrogen reduced medium, however, also seems to induce the formation of FEC after cultures have been first established on GD media [7, 45]. A gene expression analysis showed that the expression of the genes related to cell periphery, cell membrane biogenesis and cuticle biogenesis was increased and the expression of the genes related to cell-development was decreased in cassava FEC grown on the medium with reduced nitrogen, potassium, and phosphate compared with the cassava FEC grown on a CIM medium. In the current study, the frequency of FEC induction in media with reduced nitrogen, potassium and phosphate and addition of excess vitamin B1 was evaluated. Vitamin B1 is involved in the conversion from pyruvate to acetyl-CoA on energy flux as co-enzyme and it is also an important factor for plant root growth [46]. It is hypothesized that the reduced availability of nitrogen, potassium, and phosphate, and the use of excess vitamin B1 provided conditions favoring FEC induction by inhibition of non-FEC growth and promoted the structural changes of cell wall and recover energy flux by nutritional changes that helped to maintain FEC structure.

## Materials and methods

### Plant material and growth conditions

Plantlets of the ‘60444’ cassava (*Manihot esculenta* Crantz.) cultivar were obtained from the *in vitro* germplasm collection of the International Center for Tropical Agriculture (CIAT), Cali, Colombia. The *in vitro* plantlets were maintained by sub-culturing at 4–8 weeks interval on Murashige and Skoog basal salt (MS) with 20 g/L sucrose, 2 μM CuSO_4_, 2 mg/L glycine, 100 mg/L myo-Inositol, 0.5 mg/L nicotinic acid, 0.5 mg/L pyridoxine·HCI and 0.1 mg/L vitamin B1, and 3 g/L gelrite which were adjusted to pH 5.8 using 1M KOH before autoclaving for 20 min at 121°C [47]. After the five stem of *in vitro* plantlets were planted on 100 mL of fresh media, the *in vitro* plantlets were incubated at 28°C under a 16h light/8h dark photoperiod with the 40 μmol m-2 s-1. GD medium with vitamin [45] and Driver and Kuniyuki Walnut media (DKW) [48] used in this report was purchased from Duchefa. Schenk and Hildebrandt (SH) Basal Salt Mixture [49] McCown's Woody Plant Basal Salt Mixture [50], Chu (N6) Basal Salt Mixture [51] and Gamborg's B-5 Basal Salt Mixture [52] were purchased from SigmaAldrich. The chemical composition of McCow'n woody plant basal salt, Chu N6 basal salt and Gamborg B5 basal salt were shown in S1 Table.

### Production of somatic embryos (SE)

The formation of somatic embryos (SE) was induced at 2 weeks from axillary buds (AB) in a section of nodal stem of *in vitro* plantlets over a 2-month period. In order to induce the formation of the AB, all leaves were removed from the *in vitro* plantlets (length 20–30 mm), and 20–30 mm sections of single-node-stems were prepared. The single-nodal-stems were placed horizontally on petri dishes containing Murashige and Skoog basal salts (MS basal salts) with 2 μM CuSO_4_, 10 mg/L of 6-benzylaminopurine (BAP), 2% sucrose, 10 g/L Noble-agar, 2 mg/L glycine, 100 mg/L myo-Inositol, 0.5 mg/L nicotinic acid, 0.5 mg/L pyridoxine·HCI and 0.1 mg/L vitamin B1 (cassava axillary medium, CAM) (S1 Table and step 2 of S1 Fig) and incubated for 4–7 days at 28°C in the dark. The developed AB on the CAM medium were removed from the single-nodal-stems using a sterile syringe and incubated on MS basal salts with 12 mg/L picloram, 2% sucrose, 10 g/L Noble-agar, 2 mg/L glycine, 100 mg/L myo-Inositol, 0.5 mg/L nicotinic acid, 0.5 mg/L pyridoxine·HCI and 0.1 mg/L vitamin B1 (callus induction medium, CIM) for 2 weeks at 28°C in the dark to induce the formation of SE (S1 Table and step 3 of S1 Fig). The SEs subsequently were transferred to DKW media, and incubated for 2 weeks at 28°C in the dark (S1 Table and step 4 of S1 Fig).

### Production of friable embryogenic callus (FEC)

The formation of friable embryogenic callus (FEC) was induced from SE. SE were transferred to a modified MS basal salts with 2 μM CuSO_4_, 12 mg/L picloram, 2% sucrose, 10 g/L Noble-agar, 2 mg/L glycine, 100 mg/L myo-Inositol, 0.5 mg/L nicotinic acid, 0.5 mg/L pyridoxine·HCI and 10 mg/L vitamin B1 (FEC Induction Medium, FIM)(S1 Table). FIM was composed of CIM with reduced contents of nitrogen, phosphate, and potassium and an addition of 10 mg/L vitamin B1 (detailed composition provided in S1 Table and step 5 of S1 Fig). CIM-Ρ NPK was composed of CIM with reduced contents of nitrogen, phosphate, and potassium and an addition of 0.1 mg/L vitamin B1, and it was used for the FEC induction and analysis of gene expression. The SE were incubated for 2–3 weeks at 28°C in the dark. Cultures on FIM were transferred every 2–3 weeks for a maximum of six months.

### *Agrobacterium* transformation

The pCAMBIA1303 plasmid vector was electroporated into the EHA105 strain of *Agrobacterium tumefaciens* [53]. The pCAMBIA vector contains *gusA*:*gfp* genes driven by a *CaMV35S* promoter, and a hygromycin resistance gene (*HPTII*) for selection. The bacterial culture was performed as described by Bull et al. [14]. FECs were collected onto plastic plate and then the fresh weight was measured (Step 6 of S1 Fig). The FECs (fresh weight:1–2 g) were kept in FIM liquid media until *Agrobacterium* suspension was prepared. After *Agrobacterium* suspension was collected by centrifugation (4,000 x g, 28°C, 10 min), precipitation was re-suspended by 50 mL of FIM liquid media including 200 μM acetosyringone. The FECs were transferred to tea filter (30 mesh)(shown in Step 6 of S1 Fig) and the excess liquid media in FEC was removed using sterilized Kimtowel. Tea filter with FEC was soaking in a 30–40 ml of FIM liquid media including 200 μM acetosyringone with *Agrobacterium* for 2 min in petri dish while mixing gently using tweezers. After the tea filter with soaked FEC was transferred to sterilized Kimtowel, the excess *Agrobacterium* suspension was removed using the sterilized Kimtowel (Step 7 of S1 Fig). The inoculated FEC was co-cultivated on the FIM media with 200 μM acetosyringone at 24°C in the dark for 4 days (shown in Step 8 of S1 Fig). At the end of the co-cultivation period, the co-cultivated FEC was washed in FIM liquid media until the supernatant was clear. The co-cultivated FEC was kept in dark at 28°C for 4 days on FIM with 200 mg/L carbenicillin for recovery from stress of co-cultivation (shown in Step 9 of S1 Fig).

The inoculated FEC on FIM containing 200 mg/L carbenicillin was transferred to sterilized filter paper and subsequently to fresh FIM medium containing with 200 mg/L carbenicillin and a 10 mg/L of hygromycin (Step 10 of S1 Fig). The inoculated FEC cultures were maintained under a 16 h light /8 h dark photoperiod at 28°C for 7 days for the selection of transformed and untransformed FEC. A second selection was performed after another incubation period of 7 days at 28°C on FIM amended with 15 mg/L hygromycin and 200 mg/L carbenicillin (Step 11 of S1 Fig). A third selection was subsequently performed on FEC growing on FIM amended with 20 mg/L hygromycin (Step 12 of S1 Fig). The selected FEC was transferred to sterilized filter paper and then on SE emerging medium (MSN) composed of MS medium amended with 0.1 mg/L indole-3-acetic acid (NAA), 100 mg/L carbenicillin, and a 20 mg/L hygromycin for continued selection and to induce the regeneration of cotyledons (Step 13 of S1 Table). Cultures were kept at 28°C under a 16 h light/8 h dark photoperiod for 4 months, which included transfers every 2–3 weeks to fresh MSN amended with 100 mg/L carbenicillin and 20 mg/L hygromycin. The cotyledon cultures were moved to a shoot elongation medium (CEM) that was composed of MS medium amended with 0.4 mg/L of BAP, and 100 mg/L carbenicillin and 20 mg/L hygromycin (Step 14 of S1 Fig). The cotyledon cultures were maintained on CEM, with transfers to fresh CEM at 2–3 week intervals, at 28°C under a 16 h light/8 h dark photoperiod until shoots were produced. The regenerated plantlets were maintained on MS media amended with 50 mg/L of carbenicillin and 20 mg/L of hygromycin (Step 15 of S1 Fig).

### Microscopic observation

The fluorescence of green fluorescence protein (GFP) in transgenic cassava plants was observed using a BX60 fluorescence microscope (OLYMPUS, Japan).

### Purification of genomic DNA from transgenic cassava

The first or second healthy leaves from the top were used for the DNA preparation. DNA sample was extracted from 3 mm^2^ of the small cut leaves. After the leaf samples were disrupted by ShakeMaster and zirconia beads (Hirata Corporation), genomic DNA was extracted from leaf chips of the transgenic cassava plants according to the protocol of Wizard Magnetic 96 DNA Plant System (Promega) using BIOMEK (Beckman Coulter) for the confirmation of *gfp* and *hpt* genes in genomic DNA of transgenic cassava. The genomic DNA was stored at -80°C until further use.

### Confirmation of transgenic lines of cassava

The presence of *GFP* and *HPT* genes in the transgenic lines of cassava was confirmed by PCR analysis using Ex taq polymerase (Takara) and the gene-specific primers listed in S9 Table. The thermocylcer parameters used 95°C for 5 min; 35 cycles of 95°C for 30 sec, 60°C for 30 sec, and 72°C for 60 sec; and a final 5-min extension at 72°C. PCR products were analyzed by gel electrophoresis on a 1% (w/v) agarose gel stained with ethidium bromide.

### Total RNA extraction from FEC

Multiplied FECs were transferred to several media and kept during 4 weeks at 28°C in dark. The total RNA from FEC was purified by use of the Plant RNA reagent (Thermofisher) following RNeasy Plant Mini Kit (QIAGEN). A 0.5 mL of Plant RNA reagent was added to about 0.1 g of fresh weight of finely powdered FEC tissue that was crushed by a Multi-Beads Shocker (2,000 rpm, 15 s) and its mixture was mixed gently. After incubating for 5 minutes at room temperature, the supernatant was collected by centrifuging at 12,000 × g for 2 minutes at room temperature. The supernatant was transferred to a clean RNase-free tube. A 0.1 mL of 5 M NaCl was added to supernatant and tube was mixed by tapping. A 0.3 mL of chloroform was added to the supernatant and mixed thoroughly by inverting. The supernatant was collected by centrifugation at 12,000 x g for 10 minutes at 4°C to separate the phases. The upper aqueous phase was transferred to a clean RNase-free tube. After an equal volume of isopropyl alcohol was added to the collected aqueous phase, the mixture was incubated at room temperature for 10 minutes. After centrifuging the mixture at 12,000 x g for 10 minutes at 4°C, the precipitation was washed by 1.0 mL of 75% ethanol. After the solubilization of the precipitates in 40 μL of RNase-free water, the solution was treated with DNase I (10 U)(Takara) and RNase inhibitor (40 U) (Nippongene) in 50 μL of reaction at 37°C for 20 min. The 50 μL of solution was added to the 350 uL of RLT buffer in RNeasy Plant Mini Kit (QIAGEN) and the RNA preparation was performed as described by manual procedure. The total RNA quality was evaluated by electrophoresis with the bioanalyzer system (Agilent, USA). The total RNA was stored at -80°C until further use.

### Oligo-microarray analysis of gene expression in FEC and statistical analyses

The total RNA purified from FEC was used to evaluate gene expression with a cassava DNA oligo-microarray that included more than 30,000 probes as described by Utsumi et al. [54]. The presented gene expression data represents data collected from 4 independent biological replicates. A total of 8 expression microarray data sets were analyzed using GeneSpring GX (Agilent Technologies, USA). A seventy-five percentile normalization of the expression level was performed for all 8 samples. The normalized signal intensity was transformed into a log_2_ ratio for display and analysis. The normalized signal intensity of transcripts for each experiment was used for the statistical analysis. An analysis of variance test was conducted to determine the effect of treatment medium (CIM vs. CIM-Ρ NPK) on the level of FEC formation. Changes in gene expression were statistically analyzed by the unpaired t-test for two groups. A false discovery rate (q-value) was calculated based on an unpaired t-test [55]. The information from the oligo-DNA microarray was deposited in the Gene Expression Omnibus (GEO) of NCBI. The accession numbers are: Platform, GPL22197; Series, Agilent-034519 *Manihot esculenta* microarray; Samples, GSM2394462, GSM2394463, GSM2394464, GSM2394465, GSM2394466, GSM2394467, GSM2394468 and GSM2394469.

### Quantitative real-time RT-PCR (qPCR) analysis

First-strand cDNA synthesis was performed using ReverTra Ace qPCR Master Mix with gDNA Remover (Toyobo, Japan). After denaturing 1 μg of total RNA at 65°C for 5 min, 4 μL of DN Master Mix was added to the total RNA in a 16 μL total volume. The reaction was incubated for 5 min at 37°C to remove the genomic DNA. The reaction was added to 4 μL of RT master mix and incubated at 37°C for 15 min. The RT reaction was stopped by heating for 5 min at 98°C. The first-strand cDNA preparations were stored at −30°C until use. The qPCR analysis was performed with a StepOne Plus Real-Time qPCR System (Applied Biosystems, USA) using first-strand cDNA preparations containing cDNA, 5 μL of Fast SYBR Green Master Mix (Applied Biosystems, USA), and 0.1 μM of both forward and reverse primers in a 10 μL total volume. The gene-specific primers used for qPCR are listed in S9 Table. The sequences of all primer sets were designed using the Primer 3 program (http://primer3.sourceforge.net/). The PCR cycling conditions were as follows: 95°C for 20 s for initial denaturation, followed by 45 cycles of 95°C for 5 sec and 60°C for 30 sec. The specificity of the PCR amplification was evaluated with a melting curve analysis (from 55°C to 95°C) of the band pattern of the amplification product after the final cycle of the PCR. Each plate also incorporated a no-template control. Probes specific for the transcription factor SPN1 (cassava gene code, Manes.15G170300) were used as a reference. The qPCR data was analyzed with the ΔCT method using the reference gene. For each sample, the mRNA levels of target genes were normalized to that of the reference.

### Statistical analyses

All data are represented as means±SD from at least three biological experiments. Statistical analysis of means was assessed by analysis of variance using StatPlus 5 pro (AnalystSoft Inc. USA). Data for the optimization experiments was analyzed using One-Way ANOVA and differences among means were analyzed by Scheffé’s method at a 95% confidence level (p < 0.05) [56].

## Results

### Medium with reduced nitrogen, phosphate and potassium is effective for FEC induction

Although GD medium has been used in various studies as the standard medium for FEC induction (Table 1), the induction of SE, FEC and non-FEC depends on the components of other macronutrients [7]. In case of the production of SEs from cassava, the calcium concentration, the balance between nitrate and ammonium concentrations and the nitrogen concentration in media might be important factors for somatic embryogenesis. Because the increase of CaCl_2_ enhanced somatic embryogenesis as described by Li et al. [57]. Practically, DKW basal salts include more calcium in comparison to other media (S2 Fig). On the other hand, according to Taylor et al. [7], they reported that MS (minus NH_4_ and NO_3_) and SH basal salts were unstable condition for formation of organized embryogenic structure (OES), whereas GD media was the best media with regard to FEC induction and ½ MS and SH also gave a suitable FEC induction [7]. The frequency of FEC induction from SE on MS media with reduced nitrogen, as well as an absence of micronutrients; in addition to use of other types of media, should be evaluated in order to improve the frequency of FEC induction.

**Table 1:** List of genotypes and the composition of the media used to induce FEC in cassava.

Genotype Used	Successful Genotype for FEC Induction ^a^	Media for FEC Induction ^b^	Carbohydrate Type and Concentration	Auxin	Reference ^c^
60444 and M.COL.1505	60444 and M.COL.1505	MS, ½MS, MS(-NH_4_), MS(-NH_4_NO_3_), SH, N6, NN, GD, WPM and B6	2% sucrose	12 mg picloram/l	Taylor et al. 1996 (Nat. Biotechnol)
60444	60444	GD	2% sucrose	10 mg picloram/l	Raemakers et al. 1996 (Mol. Breeding)
60444 and MCOL22	60444	GD	2% sucrose	12 mg picloram/l	Zhang et al. 2000 (Plant Cell Rep.)
60444, Adira 4, R60, R90, Thai5, M7, Mcol22, Adira 1, L11 and Gading	60444, Adira 4, R60, R90, Thai5 and M7	GD	6% sucrose	6 mg picloram/l and 6 mg NAA/l	Raemakers et al. 2001 (Euphytica)
60444	60444	GD	6% sucrose	10 mg picloram/l	Schreuder et al. 2001 (Euphytica)
60444	60444	GD	2% sucrose	12 mg picloram/l	Zhang et al. 2003 (Transgenic Res.)
TME 13, TME 127, TME 8, TME 1, TMS I91/02327 and 60444	60444	GD	2% sucrose	12 mg picloram/l	Hankoua et al. 2006 (Afr. J. Biotechnol.)

Bujá Preta, Rosinha	Bujá Preta, Rosinha	GD	2% sucrose	12 mg picloram/l	Ibrahim et al. 2008 (Afr. J. Biotechnol.)
60444	60444	GD	2% sucrose	12 mg picloram/l	Bull et al. 2009 (Nat Protoc.)
T200, AR9-18, MTAI16, CR25-4, CM523-7, BRA1183, MCOL2261 and SM707-17	-	MS	2% sucrose	12 mg picloram/l	Rossin et al. 2010 (S. Afr. J. Bot.)
TME 3, TME 7 and TME 14	TME 3, TME 7 and TME 14	GD	2% sucrose	12 mg picloram/l	Zainuddin et al. 2012 (Plant Methods)
60444 and T200	60444 and T200	GD	2% sucrose	12 mg picloram/l	Chetty et al. 2013 (N Biotechnol.)
Ebwanatereka, Serere, Mkombozi, Kibandameno, Albert, Kibahaand, TME14 and 60444	Ebwanatereka, Serere and Kibandameno	GD with different concentrations of L-tyrosine (125, 250 and 500 μM)	2% sucrose	12 mg picloram/l	Nyaboga et al. 2013 (Frontiers in plant sci.)
Aladu, Bukalasa, Ebwanateraka and 60444	Aladu, Ebwanateraka, 60444	GD with 500 μM tyrosine, 50 ml of 10 mM stocks of either tyrosine or tryptophan	2% or 4% of sucrose or maltose	12 mg picloram/l	Apio et al. 2015 (Afr. J. Biotechnol.)
TME14	TME14	GD with 12 mg/l L-tyrosine	2% sucrose	12 mg picloram/l	Nyaboga et al. 2015 (Frontiers in plant sci.)
60444	60444	GD	2% sucrose	12 mg picloram/l	Ma et al. 2015 (Frontiers in plant sci.)

FEC, Friable Embryogenic Callus; MS, Murashige and Skoog Basal Salt; N6, Chu N6 Basal Salt; SH, Schenk and Hildebrandt Basal Salt; NN, Nitsch and Nitsch Basal Salt; GD, Gresshoff and Doy Basal Salt; WPM, McCow'n Woody Plant Basal Salt; B6, Gamborg B5 Basal Salt.

^a^ Thai5 and M7 were classified as relatively difficult lines to produce FEC. The efficiency of FEC production is about 1%.

^b^ Tyrosine was reported to be more suitable than tryptophan in inducing FEC formation (Apio. et al. 2015, Afr. J. Biotechnol.).

^c^ The manuscript (Rossin et al. 2010, S. Afr. J. Bot.) reported on the production of somatic embryos.

**Fig 1:**
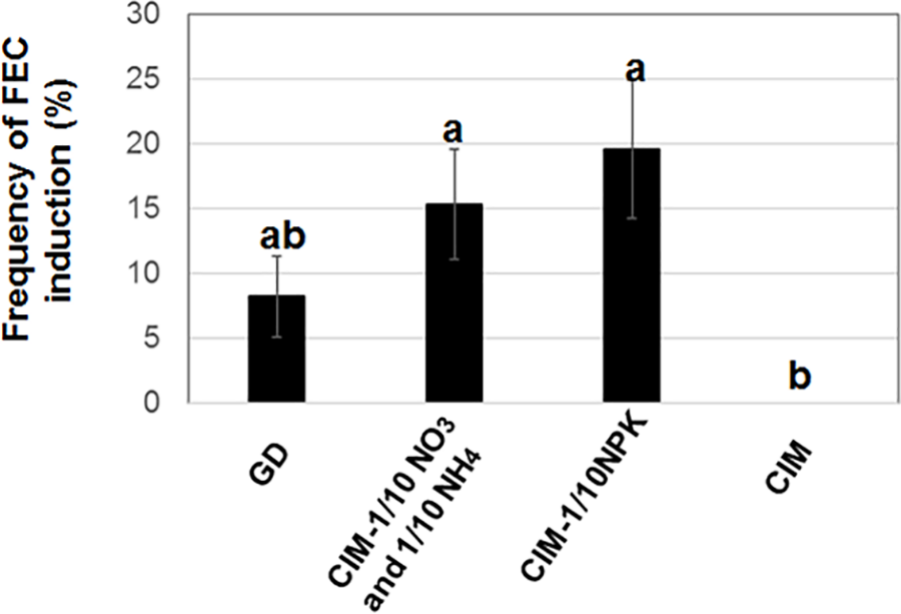
Effect of CIM-Ρ NPK on FEC production. Means and standard deviation were represented as five independent measurements as shown in S3 Table. The number of buds represented as the number of AB transferred to CIM. All FEC induced from one bud was counted as one FEC. The frequency of FEC formation was estimated as number of FEC per number of buds. FEC formation was determined as follows: The tissue like FEC and non-FEC on media were separately transferred to fresh FIM. The friable appearance as shown in Fig 8F was counted as FEC. CIM-Ρ NPK was composed of CIM with reduced content of nitrogen, phosphate, and potassium. Values labeled with different letters (a and b) at all conditions are significantly different by Scheff’s multiple comparison tests at P<0.05.

S2 Table lists the frequency of induction of FEC from SE on various types of media. Results indicated that MS (Ρ NO_3_ and Ρ NH_4_) provided the highest frequency of FEC formation. The loss of either ammonium type-nitrogen, nitrate type-nitrogen, boron or zinc from CIM negatively affected the frequency of FEC production. Chloride was included in range of 0.6–3.0 mM in all media used in this study. Subsequently, potassium and phosphate were removed from the MS (Ρ NO_3_ and Ρ NH_4_) medium, and the frequency of FEC formation was evaluated though the difference was not statistically significant (Fig 1). These results revealed that the nitrogen, phosphate and potassium-reduced CIM medium was the medium to use for producing the greatest number of FEC.

**Fig 2:**
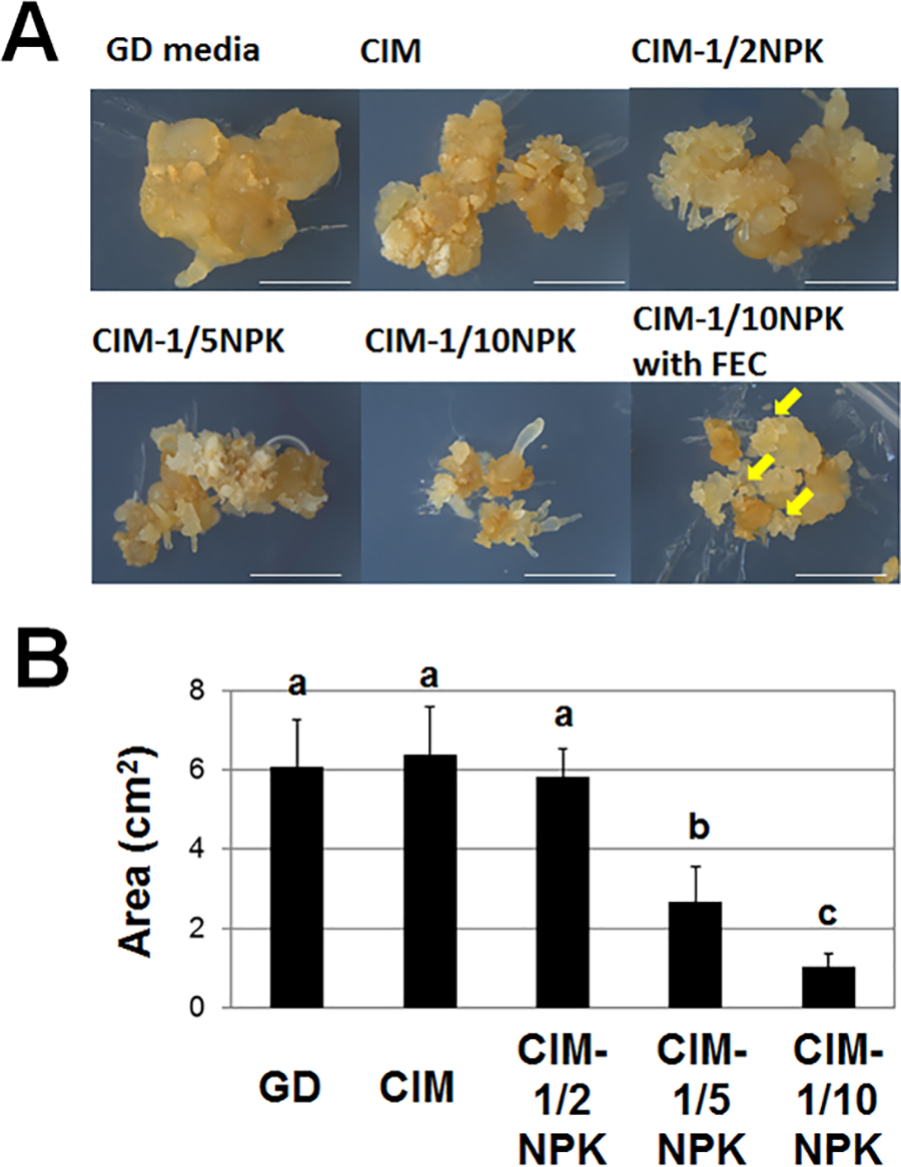
Growth of non-FEC on nitrogen, phosphate, and potassium reduced media. A) SE cultured on DKW media were grown on GD, CIM, CIM with reduced contents of nitrogen, phosphate, and potassium (CIM-½ NPK CIM-⅕ NPK, and CIM-Ρ NPK) for 3 weeks. Scale bar = 5 mm. Yellow arrows on the photo of CIM-Ρ NPK with FEC indicated the formation of FEC, B) growth area (i.e., the portion of space occupied by the callus) of the non-FEC pictured in Fig 2A was estimated after three weeks of culture. Data represent the mean ± SD as triplicate independent measurements with 10 biological replicates. Values labeled with different letters (a, b and c) at all conditions are significantly different by Scheff’s multiple comparison tests at P<0.05.

**Fig 3:**
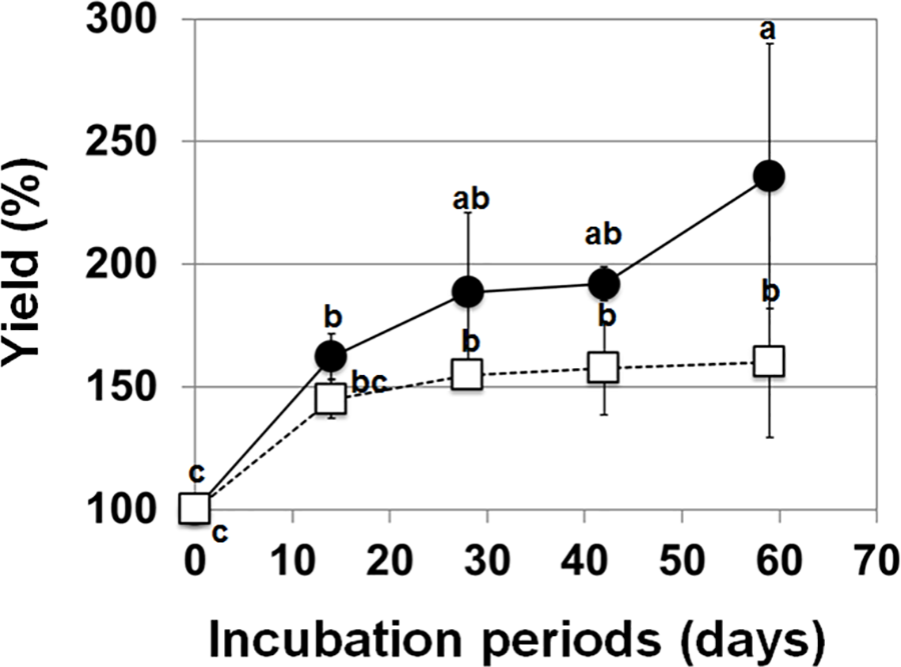
Growth profile of FEC on CIM and CIM-Ρ NPK. Growth was calculated as the percent increase in fresh weight over a 60-day period. Growth profiles of FEC on CIM (white squares) and CIM-Ρ NPK (black circles). CIM-Ρ NPK was composed of CIM with reduced content of nitrogen, phosphate, and potassium. Data represent the mean ± SD as triplicate independent measurements with two biological replicates. Values labeled with different letters (a, b and c) at all time point are significantly different by Scheff’s multiple comparison tests at P<0.05.

In order to estimate the effect on the growth, the growth area of calli was measured on CIM media containing the decreased concentrations of nitrogen, potassium and phosphate, relative to the growth area on normal CIM media and GD media (Fig 2). CIM-Ρ NPK medium inhibited the growth of non-FEC but did not affect FEC growth (Fig 3). These analyses revealed that the nitrogen, phosphate and potassium-reduced medium gave the best result among the tested ones for producing the greatest number of FEC.

### The transcripts related to cell-wall and lipid metabolism were increased on nitrogen, potassium, phosphate-reduced conditions

A cassava oligo-DNA microarray used to investigate the effect of growth medium (CIM vs. CIM-Ρ NPK) on gene expression in FEC. Total RNA isolated from FEC cultured on CIM or CIM-Ρ NPK was hybridized to the microarray and differentially expressed genes (DEGs) were identified. All microarray data were applied on the principal component analysis (PCA) and the distribution of gene expression by histogram (S3 Fig). PCA showed that the gene expression was different between FEC on CIM and FEC on CIM-Ρ NPK. Genes with an expression (FDR < 0.05 by BH method) were extracted from the resulting microarray data sets and the number of genes with two-fold higher or lower expression in FEC grown on CIM-Ρ NPK vs. CIM were determined. Expression analyses identified a total of 2,333 upregulated genes (S4 Table) and 1,515 downregulated genes (S5 Table).

**Fig 4:**
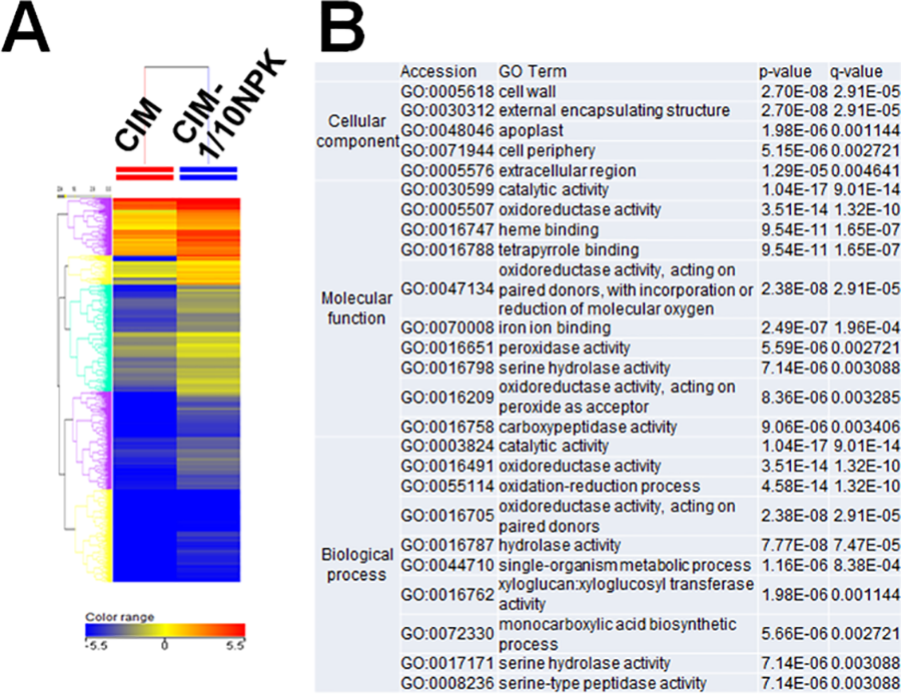
Differential gene expression in FEC cultivated on CIM-Ρ NPK by microarray analysis. (A) Heatmap for the k-means clustering of the 2,333 upregulated genes (log_2_ value>1) in FEC cultured on CIM-Ρ NPK in comparison to FEC cultured on CIM. The ratio of differential gene expression was transformed to log_2_ value. The higher and lower signal intensity values were shown in red and blue, respectively. (B) The top 10 significant gene ontology (GO) cellular process, molecular function and biological process enriched by the 2,333 upregulated genes. The *p*-value was calculated by hypergeometric test and the *q*-value was adjusted by Benjamini-Yekutieli method based on *p*-value.

The gene ontology (GO) analysis (Fig 4) and a Parametric Analysis of Gene Set Enrichment (PAGE) using agriGO software were conducted in order to identify biological processes that were altered in FEC cultured on CIM-Ρ NPK medium compared to culture on CIM media [58]. The GO terms of “cellular process”, “molecular function” and “biological process” were significantly enriched in comparison to FEC on CIM and FEC on CIM-Ρ NPK. The GO terms of “cell wall”, “external encapsulating structure”, “apoplast”, “cell periphery” in “cellular process” and “oxidoreductase activity”, “xyloglucan:xyloglucosyl transferase activity” and “monocarboxylic acid biosynthetic process” in “biological process” were significantly enriched in genes upregulated in FEC on CIM-Ρ NPK. S4 Fig illustrates the hierarchical clustering of the GO terms by agriGO (biological process) that were generated based on the genes with two-fold higher or lower expression in FEC cultured on CIM-Ρ NPK. Interestingly, among the upregulated genes, GO terms of “fatty acid metabolic process”, “membrane lipid metabolic process” and “cuticle development” related to cell-membrane development were enriched in FEC grown on CIM-Ρ NPK whereas the expression of the genes related to cell-development and cell-proliferation was decreased in cassava FEC grown on CIM-Ρ NPK.

**Fig 5:**
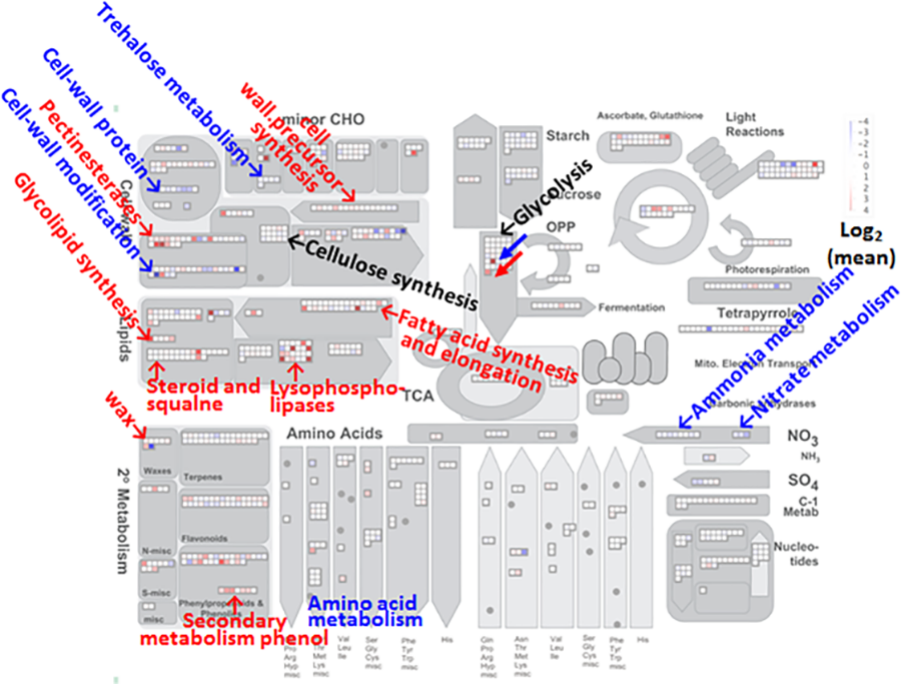
MapMan visualization of the differences in metabolism-related gene expression between FEC cultivated on CIM–Ρ NPK media and FEC cultured on CIM media. Each square represents a metabolism-related gene and displays a qualitative color code: red colors represent upregulated genes and blue colors represent downregulated genes in FEC cultured on CIM–Ρ NPK media, relative to FEC cultured on CIM media. The genes were selected from a microarray data set of comparative gene expression using the BH method (FDR <0.05) and when the gene exhibited two-fold differences (up or down) in expression as shown in S4 and S5 Tables. The means of expression data represent the average of four biological replicates. The scale of color shades represents the log_2_ values of the fold change of each of the metabolic genes. Red and blue arrows in the glycolysis metabolism section indicate PHOSPHOENOLPYRUVATE CARBOXYLASE KINASE 1 and PHOSPHOENOLPYRUVATE CARBOXYLASE KINASE 2, respectively. The blue and red words indicate GO categories that are upregulated (red) or downregulated (blue) as classified by MapMan. A black word indicates that there is no difference in this GO category.

MapMan metabolic pathways altered in FEC cultured on CIM-Ρ NPK media, relative to CIM media, are presented in Fig 5. Transcripts of several genes associated with cell-wall biosynthesis, lipid biosynthesis, and secondary metabolism were altered. These transcripts indicated to genes that encode proteins involved in the biosynthesis of cellulose precursors, pectin esterases, lipid and wax metabolisms and lignin biosynthesis (S6 Table). Collectively, the gene expression analyses indicate that transcripts involved in cell-wall and membrane production increased in FEC growing on CIM-Ρ NPK. These results suggest that changes in the expression level of these genes might contribute to the increased production of FEC during the culture on CIM-Ρ NPK.

### Addition of excess vitamin B_1_ also increased the frequency of FEC formation

**Fig 6:**
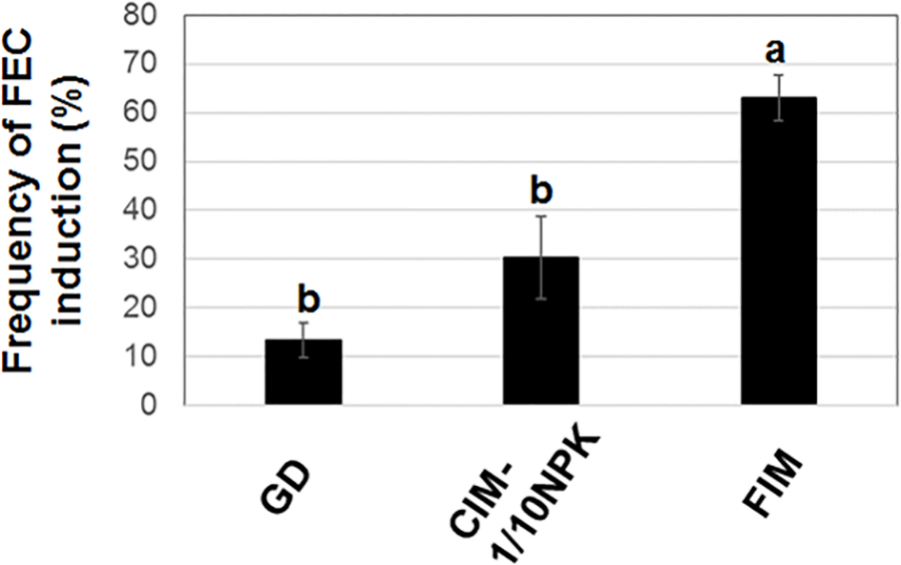
Effect of FIM on FEC production. FECs were induced on GD media, CIM-Ρ NPK and FIM. Means and standard deviation were represented as three independent measurements as shown in S7 Table. CIM-Ρ NPK was composed of CIM in which nitrogen, phosphate, and potassium was reduced. FIM was composed of CIM in which nitrogen, phosphate, and potassium was reduced and 10 mg/L vitamin B1 was added. Values labeled with different letters (a and b) at all conditions are significantly different by Scheff’s multiple comparison tests at P<0.05.

The MapMan metabolic pathways indicated that the expression of two genes encoding phosphoenolpyruvate carboxylase kinases (PPCKs) were induced in FEC cultured on CIM-Ρ NPK, relative to their expression in FEC cultured on CIM. PPCKs play a key role in the control of plant metabolism by the phosphorylation of phosphoenolpyruvate carboxylase (EC 4.1.1.31, PEPC). PEPC is mainly involved in the conversion of phosphopyruvate in glycolysis to oxalate in the TCA cycle. [59]. Vitamin B1 is involved in the conversion from pyruvate to acetyl-COA on energy production as co-enzyme and it is also one of an important factor for plant root growth [46, 60]. The energy metabolism linked from glycolysis to TCA cycle of FECs might be delay on FEC cultured on CIM-Ρ NPK media. To encourage the energy metabolism from glycolysis to TCA cycle, we attempted to add the excess vitamin B1 (0.1 mg/L to 10 mg/L) to CIM-Ρ NPK (referred as FEC Induction Medium, FIM)(Shown in M&M). The frequency of FEC formation from SE was estimated under an excess amount of vitamin B_1_ (S1 Table). Results indicated that the frequency of FEC formation was significantly higher on FIM than it was on CIM-Ρ NPK media with the normal amount of vitamin B_1_ (Fig 6). The excess vitamin B_1_ may contribute to converting pyruvate into acetyl-CoA for optimizing energy flow. Interestingly, high concentrations of vitamins are contained in commercially-produced GD media. Therefore, the use of commercial GD medium with high levels of vitamins may be the reason that GD medium supports FEC formation (S1 Table).

**Fig 7:**
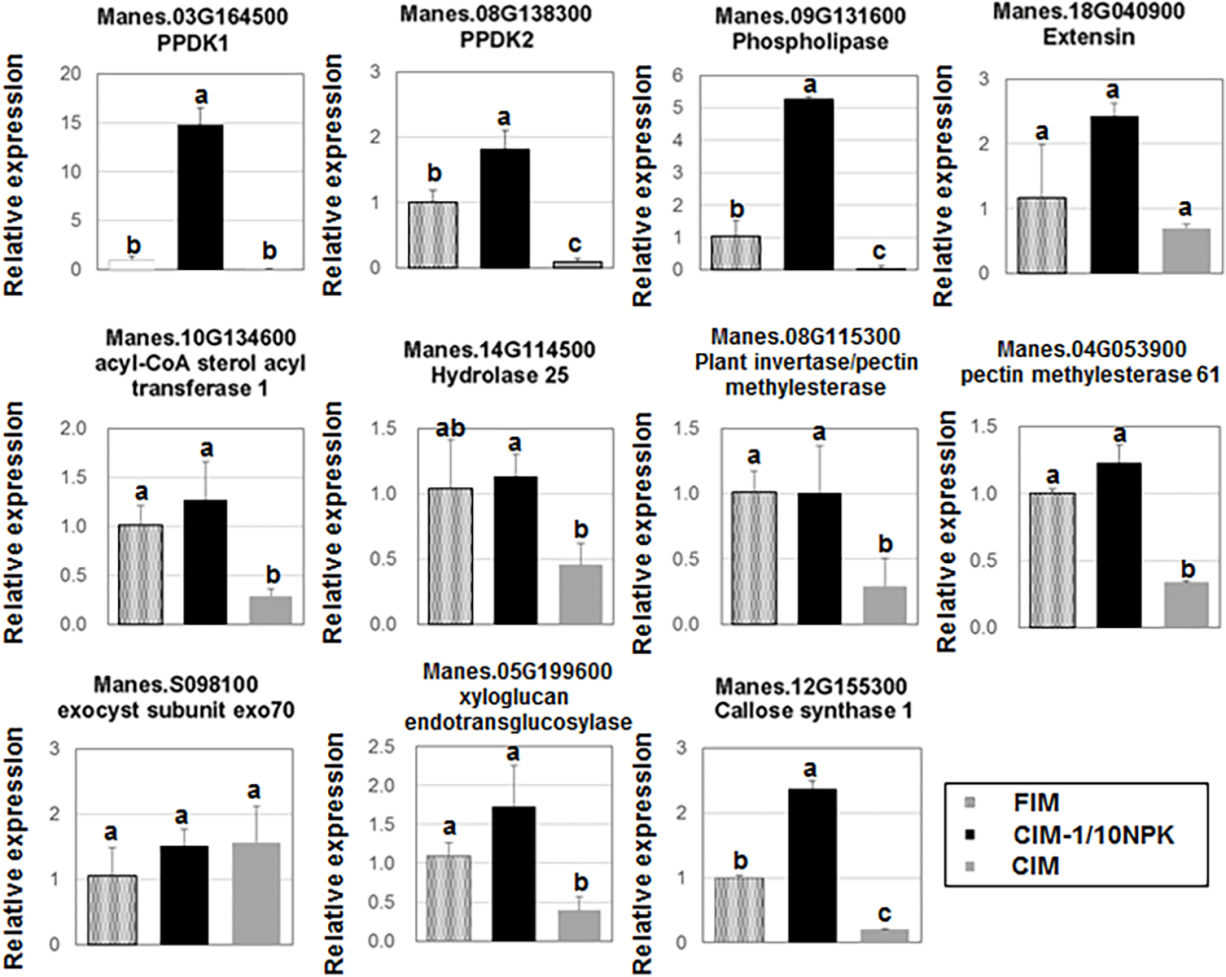
Comparison on genes associated with metabolic pathway and cellular component in FEC cultivated on three kinds of media by qRT-PCR analysis. The y-axis is relative expression level normalized against the expression level in FEC on FIM. CIM-Ρ NPK was composed of CIM in which nitrogen, phosphate, and potassium was reduced. FIM was composed of CIM in which nitrogen, phosphate, and potassium was reduced and a10 mg/L vitamin B1 was added. Means and standard deviation were represented as two independent measurements with 3 biological repeats. Values labeled with different letters (a, b and c) at all conditions are significantly different by Scheff’s multiple comparison tests at P<0.05.

To investigate the effect of vitamin B1 on FEC production, we analyzed the gene expression in FEC cultivated on three kinds of media (CIM, CIM-Ρ NPK or FIM) by qRT-PCR (Fig 7). The expression of the genes associated with GO term of “cellular component” such as plant invertase/pectin methylesterase (Manes.08G115300), pectin methylesterase 61 (Manes.04G53900), xyloglucan endotransglucosylase (Manes.05G199600) in FEC cultivated on FIM was at the same level in comparison to that of CIM-Ρ NPK, whereas transcripts of that genes on CIM were significantly decreased. The gene expression of callose synthase 1 (Manes.12G155300) in FIM was not drastically decreased in comparison to CIM-Ρ NPK. On the other hand, the gene expression of PPCK and phospholipase involved in process of energy synthesis was decreased by an addition of vitamin B1 in comparison to CIM-Ρ NPK. It might be due to that the structural changes of cell wall in FEC were not only caused by the reduction of nitrogen, potassium and phosphate and that the energy flux in FEC could be recovered by vitamin B1 addition.

### Workflow for cassava transformation

**Fig 8:**
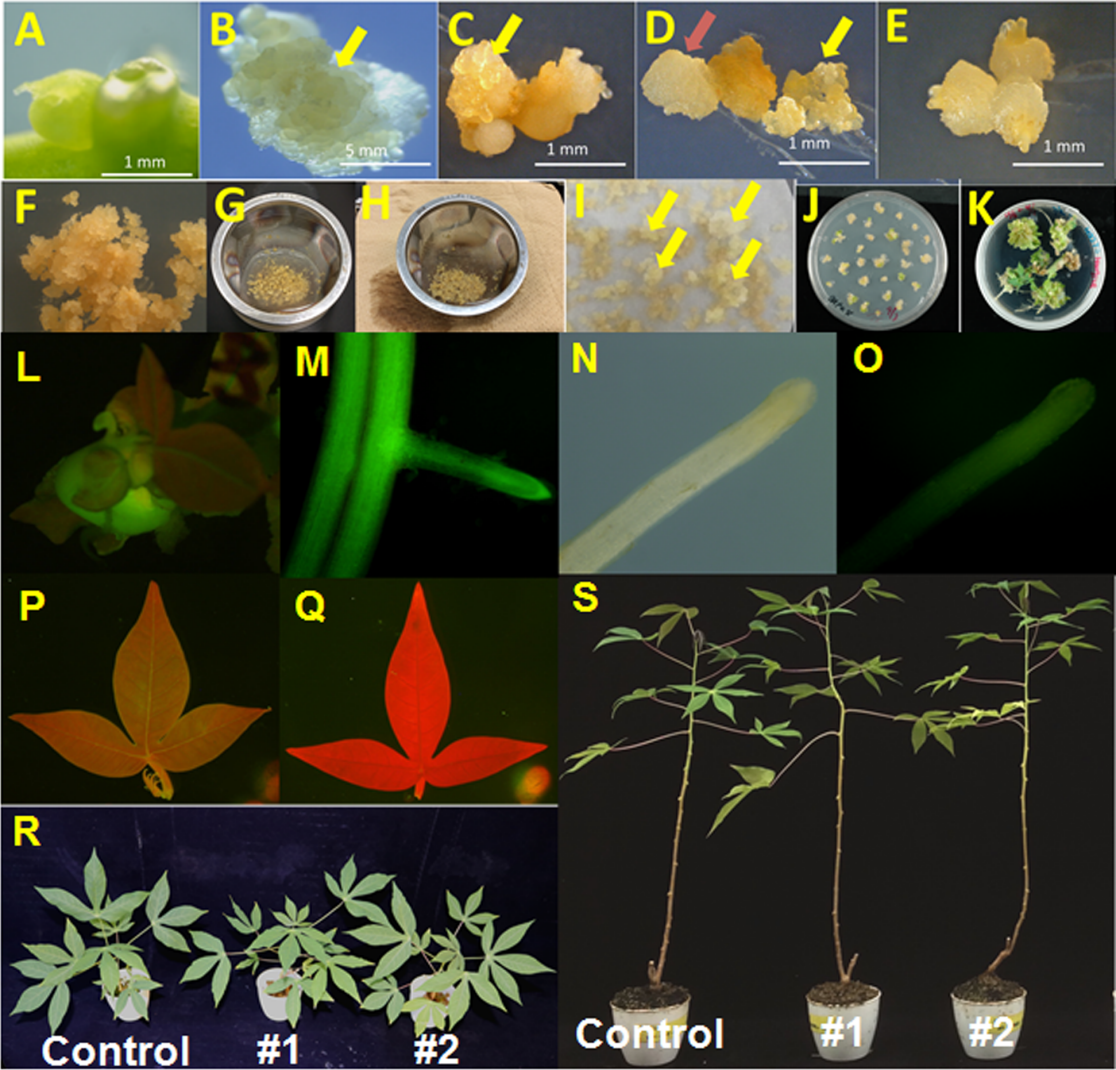
FEC induction and genetic transformation of the cassava cultivar ‘60444’. (A) AB formed in stem cuttings cultured on CAM, (B) SE produced on CIM, (C) SE produced on DKW, (D) non-FEC (red arrow) and FEC (yellow arrow) produced on CIM-Ρ NPK, (E) non-FEC cultured on FIM for an additional two weeks after transferring non-FEC in Fig 1D to fresh FIM, (F) FEC cultured on FIM for additional 4 weeks after transferring FEC in Fig 1D to fresh FIM, (G) Collection of FEC in a tea filter (30 mesh) and soaking the collected FEC in an *Agrobacterium* (EHA105) suspension for 2 min, (H) removal of excess *Agrobacterium* (EHA105) suspension by blotting on distilled Kimtowel, (I) multiplying FEC (shown in yellow arrows) on FIM amended with 20 mg/L of hygromycin, (J) induction of cotyledons from FEC on MSN amended with hygromycin, (K) induction of shoots on CEM from transferred cotyledons, (L) GFP fluorescence in transgenic *in vitro* plantlet of cassava, (M) GFP fluorescence in root tissue of a transgenic plantlet of cassava, (N) observation of root from non-transgenic *in vitro* plant using light microscope, (O) observation of root from non-transgenic *in vitro* plant using fluorescence microscope, (P) GFP fluorescence in leaves of transgenic plant, (Q) absence of GFP fluorescence in a non-transgenic cassava plant, and phenotype of transgenic (#1 and #2) and non-transgenic (control) cassava plants cultured for 2 (R) and 4 (S) months at 28°C in greenhouse after cutting.

The cassava transformation procedure using FEC is an established technique and consists of several steps (Fig 8); 1) the production of AB from stem in vitro cassava, 2) an induction of SE from AB (Fig 8A and 8B), 3) FECs induction from SE (Fig 8C–8F), 4) an inoculation with FECs and *Agrobacterium* (Fig 8G and 8H), 5) a selection of transformed FECs using media with hygromycin (Fig 8I), 6) an induction of cotyledon from selected FEC on MSN media with hygromycin and carbenicillin (Fig 8J), 7) a shoot and root formation from cotyledon on CEM media with hygromycin and carbenicillin (Fig 8K) and 8) a rooting test and a cultivation of transformants on MS media with hygromycin and carbenicillin.

The procedure of cassava transformation in this article is similar to previous method except for the media composition in a process of FEC induction, a process of infection with *Agrobacterium* and FECs and the addition of hygromycin to CEM media in the process of root and shoot formation [18, 21, 25]. In the media composition in a process of FEC induction, we used FIM for improvement of FEC induction but not GD media. In a process of infection with *Agrobacterium* and FECs, the tea filter (30 mesh) was used for the collection of FECs, the conventional infection and the removal of excess *Agrobacterium* (Fig 8G and 8H). In the process of a root and shoot formation on CEM media, CEM media with hygromycin was used for selection of the transformed cotyledon (Fig 8K). The regenerated plantlets were transferred to MS media with 10 mg/L hygromycin and 50 mg/L carbenicillin for rooting test, multiplication and management [61].

The transformants were confirmed by PCR and GFP fluorescence was observed in the transgenic lines of cassava (Fig 8L, 8M and 8P), but dull brightness and no observation in non-transgenic root and leaf, respectively in the non-transgenic, control (Fig 8O and 8Q). Fig 8R and 8S show that there were no differences between transformant and non-transformant on the vigor and other phenotypes. However, we sometimes observed that several plants regenerated from FEC after the long-time (more than 1 year) culture of FEC showed the altered phenotypes, such as shrunken leaves compared with control plants. Therefore, we recommend that the period of FEC induction should be within 1 year as described by Bull et al (2006) [14].

Finally, we estimated the efficiency of cassava transformation from 10 experiments according to process of transformation in this report. Number of regenerated plants per one gram of FEC fresh weight on FIM or on GD media was 14.7 (estimated as 10 independent experiments) or 14.8 (estimated as 5 independent experiments), respectively. We collected total 2,709 cotyledons (including non-transformants and transformants) from total 31.6 g fresh weight of FEC cultured on FIM. Among 2,709 cotyledons, 1,020 survived on CEM with hygromycin and finally 465 plantlets were regenerated. The percentage of regeneration efficiency was 45.6% (456/1,020). This was consistent with that of Bull et al. [14], reporting that the regeneration efficiency is about 40–70%.

## Discussion

In this study, we developed an improved medium for the efficient production of FEC in cassava, which is an essential need for the genetic transformation of cassava. Relative to the standard medium that is currently used, and other mediums that were evaluated, whereas the frequency of FEC formation was improved by culture on a nitrogen, phosphate, and potassium-reduced medium (CIM-Ρ NPK) and the non-FEC growth was inhibited on nitrogen, potassium and phosphate-reduced medium. Additionally, the frequency of FEC formation was also increased by the addition of excess vitamin B1 to the CIM-Ρ NPK medium (FIM). It is hypothesized that the reduced availability of nitrogen, potassium, and phosphate, and the use of excess vitamin B1 provided conditions favoring FEC induction by inhibition of non-FEC growth and promoted the structural changes of cell wall and recover of energy flux by nutritional changes that helped to maintain FEC structure.

As reasons that frequency of FEC production on FIM was increased, we think: 1) the process of FEC identification was easier by inhibition of non-FEC growth, 2) structural changes of FEC in cell wall might be promoted by decrease of nutrition in media. In this article, we observed that the non-FEC growth on decreasing nitrogen, potassium and phosphate was inhibited. The non-FEC shows in the process of rapid cell division in comparison to FEC and SE and might consume more energy [62]. On the other hand, the frequency of FEC production was improved under nutrition decreasing condition. The gene expression associated with energy flux such as sugar and lipid metabolism was recovered in FEC on FIM whereas the gene expression associated with cell wall and cell periphery was not dramatically decreased in FEC cultivated on FIM in spite of recovery of gene expression associated with energy flux. We suggest that the condition such as reduction of nitrogen, potassium and phosphate and addition of vitamin B1 are effective for FEC production in cv. 60444.

Many studies have been conducted in cassava to increase the formation of SE and FEC in order to improve the efficiency of cassava transformation. High concentrations of CaCl_2_ was found to promote SE formation but not FEC formation in cassava [57]. An increase of sucrose content (increase from 2% sucrose to 6% sucrose) in the liquid SH medium contributed to the embryogenic suspension of FEC [15, 16]. Also, an addition of tyrosine is more effective for FEC production from some cassava cultivars [25–28]. The use of diluted MS basal salts and SH media along with the addition of picloram has a positive effect for the formation of FEC as well as use of GD medium. Removal of ammonium or ammonium and nitrate from CIM media showed not only an negative effect for the frequency of non-FEC formation but also decreased FEC formation [7]. In this manuscript, although the growth of non-FEC depended on amount of nitrogen, phosphate and potassium in media, the induction of FEC was not inhibited dramatically under reduced nitrogen, phosphate and potassium in media. The changes in nutrition might lead to prevent ecotopic callus formation such as prevention of orderly deposition of cell wall polysaccharides [63].

For the transformation efficiency, transformation efficiency by our protocol was not dramatically decreased compared with that by Taylor’s method [22]. We obtained average 14.7 transgenic lines per one gram of FEC fresh weight using FEC on FIM by this protocol, whereas we prepared average 14.8 transgenic lines per one gram of FEC fresh weight using FEC on GD media (estimated as 5 independent experiments) within maximum 9 month after *Agrobacterium* inoculation with FEC. Practically, transgenic lines could be obtained within 6 months after *Agrobacterium* inoculation in most cases. Taylor et al. (2012) obtained average 22.0 transgenic plants per cm^3^ of settled cell volume (SCV) of FEC of model cultivar 60444. When one gram of FEC was converted to 0.998 g per mL of FEC volume (n = 5, 24°C), nevertheless the method of regeneration process is similar to other method, indicating that the quality of FEC was not changed between FEC on media in this report and FEC on GD media.

Bull et al. (2009) prepared 50 transgenic plants by *Agrobacterium*-mediated transformation using 100 clusters of FEC for model cultivar 60444 [14]. Zainuddin et al. (2012) obtained 7–17 transgenic lines per 18 clusters by transformation using FEC of cassava landraces [23]. Chetty et al. (2013) obtained 23 transgenic lines per 100 clusters by transformation using FEC of cassava cultivar T200 [24]. The one of SCV equivalent to 20 FEC clusters [25]. Nyaboga et al. (2015) prepared approximately 70–80 transgenic lines per ml SCV using FEC from cultivar TME14 in about 4–5 months after *Agrobacterium* inoculation with FEC [25]. Although the broad variation on transformation efficiency among each report was observed, the transformation efficiency seems to be improved by frequent exchange of cotyledon or selected FEC to fresh media of MSN and CEM [14] and/or the use of smaller pore size of tea filter in process of *Agrobacterium* infection and washing might be effective for improvement of cassava transformation because it can collect the smaller size of FECs. On the other hand, cost management must be also considered. All chemicals for preparation of FIM are obtainable at chemical companies independently. Therefore, we wish that cost for media preparation is reasonable but it might be dependent on country situation.

As current situation, it is one of important research to develop transformation methods for farmer- and industry-preferred landraces and cultivars for deployment and adoption of transgenic cassava in the field. Therefore, although we have attempted a transformation using Asian-elite cultivar KU50, we found that there is problem for FEC induction from the cultivar. It is that FEC formation was observed and the frequency of FEC induction was improved in comparison to GD media but the ability of FEC multiplication was quite low. To develop the cassava breeding in Asian countries, we continuously investigate to optimize the condition for multiplying FECs from Asian elite cultivars.

Transformation technology for several industry-preferred African cultivars of cassava has been developed, however, few reproducible transformation technology has been developed for industry-preferred Asian cultivars of cassava, because the frequency of FEC induction is quite low and a protocol for the reproducible FEC production has not been established in most Asian cultivars. Although the transformation via SE for Asian cultivars has already been reported, the transgenic lines obtained were likely genetically unstable plants and/or chimeric transgenic plants [20]. As next step, we plan to apply the optimized protocol developed in the current study to the transformation of farmer- and industry-preferred cultivars in Asia. The recently developed protocol, that significantly increases FEC formation in cassava, is expected to greatly benefit the use of genetic engineering technologies to improve the stress and disease resistance, as well as tuber quality, of cassava genotypes adapted to local environments in Asia.

## Supporting information

S1 Fig Workflow of *Agrobacterium*-mediated transformation of cassava. The present study was modified on the culture conditions in step 5.
(TIF)

S2 Fig **The concentration of ammonium (A), nitrogen (B), phosphate (C), potassium (D), boron (E), calcium (F), magnesium (G), sulfur (H), Cl (Cl), microelements (J), and other microelements (K and L) used in the various media.** MS, DKW, GD SH, McCW, Chu and GB5 indicate Murashige and Skoog medium [47], Driver-Kuniyuki Walnut medium [48], Gresshoff and Doy medium [45], Schenk and Hildebrandt (SH) Basal Salt Mixture [49] McCown's Woody Plant Basal Salt Mixture [50], Chu (N6) Basal Salt Mixture [51] and Gamborg's B-5 Basal Salt Mixture [52], respectively.
(TIF)

S3 Fig **Principal component analysis of the gene expression identified by microarray (A), Frequency histogram of gene expression in microarray. The X-axis shows the normalized gene expression level. The Y-axis shows the number of genes at a given expression level (B).** Red and blue circles on S2 Fig. A showed the FEC on CIM and the FEC on CIM-Ρ NPK, respectively.
(TIF)

S4 Fig Hierarchical clustering of GO terms enriched in FEC cultivated on CIM-Ρ NPK media relative to FEC cultured on CIM media. The graph was generated for the GO terms within the category biological process by Parametric Analysis of Gene Set Enrichment (PAGE) in agriGO [58]. A total of 10,959 genes, selected using the BH method (FDR<0.05), and annotated with an Arabidopsis gene code, were used in the analysis. The first pair of numbers represent the number of genes in the input list associated with the GO term and the number of genes in the input list, respectively. The second pair of numbers represent the number of genes associated with the GO term in the Arabidopsis database (TAIR9) and the total number of Arabidopsis genes with GO annotations in TAIR9. Box colors indicate the levels of statistical significance (FDR < 0.1 as determined using the Hochberg FDR).
(TIF)

S1 Table Chemical composition of the various media used in the current study. (XLSX)

S2 Table Effect of various kinds of media on the induction of friable embryogenic callus (FEC) formation in cassava. (XLSX)

S3 Table Effect of nitrogen, phosphate, and potassium reduced media on FEC production shown in Fig 1. (XLSX)

S4 Table List of genes upregulated (>two-fold) in FEC cultured on CIM-Ρ NPK vs. CIM media. (XLSX)

S5 Table List of genes downregulated (<two-fold) in FEC cultured on CIM-Ρ NPK vs. CIM media. (XLSX)

S6 Table List of differentially expressed genes (log2FC < -1 and log2FC > 1) in FEC cultured on CIM-Ρ NPK vs. CIM media by agriGO. (XLSX)

S7 Table Effect of media, including 0.1 mg/L of vitamin B1 or 10 mg/L of vitamin B1, on the induction of FEC formation shown in Fig 6. (XLSX)

S8 Table List of reported genes implicated in callus induction or repression in Arabidopsis. (XLSX)

S9 Table List of gene-specific primers used in this study. (XLSX)

## References

[1] Balat M, Balat H. Recent trends in global production and utilization of bio-ethanol fuel. Applied Energy. 2009;86: 2273–2282.

[2] Jansson C, Westerbergh A, Zhang J, Hu X, Sun C. Cassava, a potential biofuel crop in (the) People’s Republic of China. Applied Energy. 2009;86: S95–S99.

[3] Ferraro V, Piccirillo C, Tomlins K, Pintado ME. Cassava (*Manihot esculenta* Crantz) and Yam (*Dioscorea* spp.) crops and their derived foodstuffs: safety, security and nutritional value. Critical Reviews Food Sci Nutrit. 2015;56: 2714–2727. 10.1080/10408398.2014.922045 26165549

[4] Ceballos H, Iglesias CA, Pérez JC, Dixon AGO. Cassava breeding: opportunities and challenges. Plant Mol Biol. 2004;56: 503–516. doi: 10.1007/s11103-004-5010-5 15630615 10.1007/s11103-004-5010-5

[5] Liu J, Zheng Q, Ma Q, Gadidasu KK, Zhang P. Cassava genetic transformation and its application in breeding. J Integr Plant Biol. 2011;535: 52–69. 10.1111/j.1744-7909.2011.01048.x 21564542

[6] Stamp JA, Henshaw GG. Somatic embryogenesis in cassava. Z Pflanzenphysiol. 1982;105: 183–187.

[7] Talyor NJ, Edwards M, Kiernan RJ, Davey CDM, Blakesley D, Henshaw GG. Development of friable embryogenic callus and embryogenic suspension culture systems in cassava (*Manihot esculenta* Crantz). Nat Biotechnol. 1996; 726–730. doi: 10.1038/nbt0696-726 9630979 10.1038/nbt0696-726

[8] Li H-Q, Sautter C, Potrykus I, Puonti-Kaerlas J. Genetic transformation of cassava (*Manihot esculenta* Crantz). Nat Biotechnol. 1996;14: 736–740. doi: 10.1038/nbt0696-736 9630981 10.1038/nbt0696-736

[9] Schöpke C, Taylor N, Cárcamo R, N.K. K, Marmey P, Henshaw GG, et al Regeneration of transgenic cassava plants (*Manihot esculenta* Crantz) from microbombarded embryogenic suspension cultures. Nat Biotechnol. 1996;14: 731–735. doi: 10.1038/nbt0696-731 9630980 10.1038/nbt0696-731

[10] Zhang P, Legris G, Coulin P, Puonti-Kaerlas J. Production of stably transformed cassava plants via particle bombardment. Plant Cell Rep. 2000;19: 939–945. 10.1007/s002990000224 30754836

[11] Raemakers CJJM, Sofiari E, Taylor N, Henshaw G, Jacobsen E, Visser RGF. Production of transgenic cassava (*Manihot esculenta* Crantz) plants by particle bombardment using luciferase activity as selection marker. Mol Breed. 1996;2: 339–349.

[12] González AE, Schöpke C, Taylor NJ, Beachy RN, Fauquet CM. Regeneration of transgenic cassava plants (*Manihot esculenta* Crantz) through *Agrobacterium*-mediated transformation of embryogenic suspension cultures. Plant Cell Rep. 1998;17: 827–831. 10.1007/s002990050492 30736551

[13] Zhang P and Gruissem W. Production of transgenic cassava (*Manihot esculenta* Crantz) in Transgenic Crops of the World—Essential Protocols. Kluwer Academic Publishers 2004; 301–319.

[14] Bull SE, Owiti JA, Niklaus M, Beeching JR, Gruissem W, Vanderschuren H. *Agrobacterium*-mediated transformation of friable embryogenic calli and regeneration of transgenic cassava. Nat Protocol. 2009;4: 1845–1854. 10.1038/nprot.2009.208 20010938

[15] Raemakers K, Schreuder M, Pereira I, Munyikwa T, Jacobsen E, Visser R. Progress made in FEC transformation of cassava. Euphytica. 2001;120: 15–24.

[16] Schreuder MM, Raemakers CJJM, Jacobsen E, Visser RGF. Efficient production of transgenic plants by *Agrobacterium*-mediated transformation of cassava (*Manihot esculenta* Crantz). Euphytica. 2001;120: 35–42.

[17] Taylor NJ, Masona MV, Carcamo R, Ho T, Schöpke C, Fauquet CM. Production of embryogenic tissues and regeneration of transgenic plants in cassava (*Manihot esculenta* Crantz). Euphytica. 2001;120: 25–34.

[18] Zhang P, Potrykus I, Puonti-Kaerlas J. Efficient production of transgenic cassava using negative and positive selection. Transgenic Res. 2000;9: 405–415. 11206969 10.1023/a:1026509017142

[19] Saelim L, Phansiri S, Netrphan S, Suksangpanomrung M, Narangajavana J. Optimization of In vitro cyclic somatic embryogenesis and regeneration of the Asian cultivars of cassava (*Manihot esculenta* Crantz) for genetic manipulation system. Global J Biotechnol Biochem Res. 2006;1: 7–15.

[20] Saelim L, Phansiri S, Suksangpanomrung M, Netrphan S, Narangajavana J. Evaluation of a morphological marker selection and excision system to generate marker-free transgenic cassava plants. Plant Cell Rep. 2009;28: 445–55. doi: 10.1007/s00299-008-0658-y 19093119 10.1007/s00299-008-0658-y

[21] Sayre R, Beeching JR, Cahoon EB, Egesi C, Fauquet C, Fellman J, Fregene M, Gruissem W, Mallowa S, Manary M, Maziya-Dixon B, Mbanaso A, Schachtman DP, Siritunga D, Taylor N, Vanderschuren H, Zhang P. The BioCassava plus program: biofortification of cassava for sub-Saharan Africa. Annu Rev Plant Biol. 2011; 62:251–272. doi: 10.1146/annurev-arplant-042110-103751 21526968 10.1146/annurev-arplant-042110-103751

[22] Taylor N, Gaitán-Solís E, Moll T, Trauterman B, Jones T, Pranjal A, Trembley C, Abernathy V, Corbin D, Fauquet CM. A High-throughput platform for the production and analysis of transgenic cassava (*Manihot esculenta*) plants. Tropical Plant Biol. 2012; 5:127–139

[23] Zainuddin IM, Schlegel K, Gruissem W, Vanderschuren H. Robust transformation procedure for the production of transgenic farmer-preferred cassava landraces. Plant Meth. 2012;8: 24. 10.1186/1746-4811-8-24 PMC3439245 22784378

[24] Chetty CC, Rossin CB, Gruissem W, Vanderschuren H, Rey ME. Empowering biotechnology in southern Africa: establishment of a robust transformation platform for the production of transgenic industry-preferred cassava. N Biotechnol. 2012;25: 136–143. 10.1016/j.nbt.2012.04.006 22683498

[25] Nyaboga E, Njiru J, Nguu E, Gruissem W, Vanderschuren H, Tripathi L. Unlocking the potential of tropical root crop biotechnology in east Africa by establishing a genetic transformation platform for local farmer-preferred cassava cultivars. Front Plant Sci. 2013;4: 526 doi: 10.3389/fpls.2013.00526 24400011 10.3389/fpls.2013.00526 PMC3872047

[26] Chauhan RD, Beyene G, Kalyaeva M, Fauquet CM, Taylor N. Improvements in *Agrobacterium*-mediated transformation of cassava (*Manihot esculenta* Crantz) for large-scale production of transgenic plants. Plant Cell Tiss Organ Cult. 2015; 121:591–603.

[27] Apio HB, Alicai T, Baguma Y, Mukasa SB, Bua A, Taylor N. Production of friable embryogenic callus and regeneration of Ugandan farmer-preferred cassava genotypes. Afr J Biotechnol. 2015;14: 1854–1864.

[28] Nyaboga EN, Njiru JM, Tripathi L. Factors influencing somatic embryogenesis, regeneration, and *Agrobacterium*-mediated transformation of cassava (*Manihot esculenta* Crantz) cultivar TME14. Front Plant Sci. 2015;6: 411 doi: 10.3389/fpls.2015.00411 26113851 10.3389/fpls.2015.00411 PMC4461822

[29] Zhang P., Vanderschuren H., Fütterer J. & Gruissem W. Resistance to cassava mosaic disease in transgenic cassava expressing antisense RNAs targeting virus replication genes. Plant Biotechnol J. 2005;3: 385–397. doi: 10.1111/j.1467-7652.2005.00132.x 17173627 10.1111/j.1467-7652.2005.00132.x

[30] Vanderschuren H, Akbergenov R, Pooggin MM, Hohn T, Gruissem W, Zhang P. Transgenic cassava resistance to African cassava mosaic virus is enhanced by viral DNA-A bidirectional promoter-derived siRNAs. Plant Mol Biol. 2007;64: 549–557. doi: 10.1007/s11103-007-9175-6 17492253 10.1007/s11103-007-9175-6

[31] Ntui VO, Kong K, Khan RS, Igawa T, Janavi GJ, Rabindran R, Nakamura I, Mii M. Resistance to Sri Lankan Cassava Mosaic Virus (SLCMV) in Genetically Engineered Cassava cv. KU50 through RNA Silencing. PLoS One. 2015;10:e0120551 doi: 10.1371/journal.pone.0120551 25901740 10.1371/journal.pone.0120551 PMC4406713

[32] Jitenders Y, Ogwok E, Wagaba H, Patil BL, Bagewadi B, Alicai T, et al RNAi-mediated resistance to Cassava brown streak Uganda virus in transgenic cassava. Mol Plant Pathol. 2011;12: 677–687. doi: 10.1111/j.1364-3703.2010.00700.x 21726367 10.1111/j.1364-3703.2010.00700.x PMC6640337

[33] Zhang P, Wang WQ, Zhang GL, Kaminek M, Dobrev P, Xu J, et al Senescence-inducible expression of isopentenyl transferase extends leaf life, increases drought stress resistance and alters cytokinin metabolism in cassava. J Integr Plant Biol. 2010;52: 653–669. doi: 10.1111/j.1744-7909.2010.00956.x 20590995 10.1111/j.1744-7909.2010.00956.x

[34] Zidenga T, Leyva-Guerrero E, Moon H, Siritunga D, Sayre R. Extending cassava root shelf life via reduction of reactive oxygen species production. Plant Physiol. 2012;159: 1396–1407. doi: 10.1104/pp.112.200345 22711743 10.1104/pp.112.200345 PMC3425186

[35] Owiti J, Grossmann J, Gehrig P, Dessimoz C, Laloi C, Hansen M B, Gruissem W, Vanderschuren H. iTRAQ-based analysis of changes in the cassava root proteome reveals pathways associated with post-harvest physiological deterioration. Plant J. 2011;67: 145–156. doi: 10.1111/j.1365-313X.2011.04582.x 21435052 10.1111/j.1365-313X.2011.04582.x

[36] Xu J, Duan X, Yang J, Beeching JR, Zhang P. Enhanced ROS scavenging by over-production of superoxide dismutase and catalase delays post-harvest physiological deterioration of cassava storage Roots. Plant Physiol. 2013;161: 1517–1528. doi: 10.1104/pp.112.212803 23344905 10.1104/pp.112.212803 PMC3585613

[37] Welsch R, Arango J, Bar C, Salazar B, Al-Babili S, Beltran J, et al Provitamin A accumulation in cassava (*Manihot esculenta*) roots driven by a single nucleotide polymorphism in a phytoene synthase gene. Plant Cell. 2010;22: 3348–3356. doi: 10.1105/tpc.110.077560 20889914 10.1105/tpc.110.077560 PMC2990137

[38] Li KT, Moulin M, Mangel N, Albersen M, Verhoeven-Duif NM, Ma Q, et al Increased bioavailable vitamin B6 in field-grown transgenic cassava for dietary sufficiency. Nat Biotechnol. 2015;33: 1029–1032. 10.1038/nbt.3318 26448082

[39] Jorgensen K, Bak S, Busk PK, Sorensen C, Olsen CE, Puonti-Kaerlas J, et al Cassava plants with a depleted cyanogenic glucoside content in leaves and tubers. Distribution of cyanogenic glucosides, their site of synthesis and transport, and blockage of the biosynthesis by RNA interference technology. Plant Physiol. 2005;139: 363–374. doi: 10.1104/pp.105.065904 16126856 10.1104/pp.105.065904 PMC1203385

[40] Ihemere U, Arias-Garzon D, Lawrence S, Sayre R. Genetic modification of cassava for enhanced starch production. Plant Biotechnol J. 2006;4: 453–465. doi: 10.1111/j.1467-7652.2006.00195.x 17177810 10.1111/j.1467-7652.2006.00195.x

[41] Zhao SS, Dufour D, Sánchez T, Ceballos H, Zhang P. Development of waxy cassava with different Biological and physico-chemical characteristics of starches for industrial applications. Biotechnol Bioeng. 2011;108:1925–1935. doi: 10.1002/bit.23120 21370230 10.1002/bit.23120

[42] Koehorst-van Putten HJ, Sudarmonowati E, Herman M, Pereira-Bertram IJ, Wolters AM, Meima H, et al Field testing and exploitation of genetically modified cassava with low-amylose or amylose-free starch in Indonesia. Transgenic Res. 2012;21: 39–50. doi: 10.1007/s11248-011-9507-9 21465166 10.1007/s11248-011-9507-9 PMC3264866

[43] Ihemere UE, Narayanan NN, Sayre R. Iron biofortification and homeostasis in transgenic cassava roots expressing the algal iron assimilatory gene, *FEA1*. Front Plant Sci. 2012;3:171 doi: 10.3389/fpls.2012.00171 22993514 10.3389/fpls.2012.00171 PMC3440605

[44] Chavarriaga-Aguirre P, Brand A, Medina A, Prías M, Escobar R, Martinez J, Díaz P, López C, Roca WM, Tohme J. The potential of using biotechnology to improve cassava: a review. In Vitro Cell Dev Biol Plant. 2016; 52: 461–478. doi: 10.1007/s11627-016-9776-3 27818605 10.1007/s11627-016-9776-3 PMC5071364

[45] Gresshoff PM, Doy CH. Development and differentiation of haploid Lycopersicon esculentum (tomato). Planta. 1972;107: 161–170. doi: 10.1007/BF00387721 24477400 10.1007/BF00387721

[46] Booner J, Green J. Vitamin B1 and the growth of green plants. Bot Gaz. 1938;100: 226–237.

[47] Murashige T, Skoog S. A revised medium for rapid growth and bioassays with tabacco tissue cultures. Physiol Plantarum. 1962;15: 473–497.

[48] Driver J, Rodriguez R, Kuniyuki AH. In vitro propagation of paradox walnut rootstock (*Juglans hindsii* x *J*. *regia*). HortScience 1981;19: 507–509.

[49] Schenk RU, Andhildebrandt AC. Mediunland techniques forinduction and growth of monocotyledonous and dicotyledonous plant cell cultures. Can J Bot. 1972;50:199–204.

[50] Lloyd G, McCown B. Commercially feasible micropropagation of mountain laurel, *Kalmia latifolia*, by use of shoot tip culture. Proc Int Plant Prop Soc. 1980;30: 421–427.

[51] Chu CC. Establishment of an efficient medium for anther culture of rice, through comparative experiments on the nitrogen sources. Sci Sin. 1975;18: 659–668.

[52] Gamborg OL, Miller RA, Ojima K. Nutrient requirements of suspension cultures of soybean root cells. Exp Cell Res. 1968;50: 151–158. 5650857 10.1016/0014-4827(68)90403-5

[53] Hood EG, Chilton WS, Chilton M-D, Fraley RT. T-DNA and opine synthetic loci in tumors incited by *Agrobacterium tumefaciens* A281 on Soybean and Alfalfa plants. J Bacteriol. 1986;168: 1283–1290. 3023301 10.1128/jb.168.3.1283-1290.1986 PMC213635

[54] Utsumi Y, Tanaka M, Kurotani A, Yoshida T, Mochida K, Matsui A, et al Cassava (*Manihot esculenta*) transcriptome analysis in response to infection by the fungus *Colletotrichum gloeosporioides* using an oligonucleotide-DNA microarray. J Plant Res. 2016;129: 711–726. doi: 10.1007/s10265-016-0828-x 27138000 10.1007/s10265-016-0828-x

[55] Benjamini Y, Hochberg Y. Controlling the false discovery rate: a practical and powerful approach to multiple testing. J R Statist Soc B. 1995;57: 289–300.

[56] Bother R. On sharpening Scheffe bounds. J Roy Stat Soc. 1967;29: 110–114.

[57] Li RM, Hu XW, Li KM, Fu SP, Guo JC. CaCl2 enhanced somatic embryogenesis in *Manihot esculenta* Crantz. Biosci Biotechnol Biochem. 2009;73: 2513–2515. doi: 10.1271/bbb.90321 19897906 10.1271/bbb.90321

[58] Du Z, Zhou X, Ling Y, Zhang Z, Su Z. agriGO: a GO analysis toolkit for the agricultural community. Nucleic Acids Res. 2010;38: W64–70. doi: 10.1093/nar/gkq310 20435677 10.1093/nar/gkq310 PMC2896167

[59] Hartwell J, Gill A, Nimm GA, Wilkin MB, Jenkins GI, Nimmo HG. Phosphoenolpyruvate carboxylase kinase is a novel protein kinase regulated at the level of expression. Plant J. 1999;20: 333–342. 10571893 10.1046/j.1365-313x.1999.t01-1-00609.x

[60] Dhillon RS, Hooda MS, Pundeer JS, Ahlawat KS, Chopra AI. Effects of auxins and thiamine on the efficacy of techniques of clonal propagation in *Jatropha curcas* L. Biomass and Bioenergy. 2011;35: 1502–1510.

[61] Zhang P, Potrykus I, Puonti-Kaerlas J. Efficient production of transgenic cassava using negative and positive selection. Transgenic Res. 2000;9: 405–415. 11206969 10.1023/a:1026509017142

[62] Ma Q, Zhou W, Zhang P. Transition from somatic embryo to friable embryogenic callus in cassava: dynamic changes in cellular structure, physiological status, and gene expression profiles. Front Plant Sci. 2015;6: 824 doi: 10.3389/fpls.2015.00824 26500668 10.3389/fpls.2015.00824 PMC4594424

[63] Ikeuchi M, Sugimoto K, Iwase A. Plant callus: mechanisms of induction and repression. Plant Cell. 2013;25: 3159–3173. doi: 10.1105/tpc.113.116053 24076977 10.1105/tpc.113.116053 PMC3809525

