# Supplementary figures and images for: Formation of friable embryogenic callus in cassava is enhanced under conditions of reduced nitrate, potassium and phosphate
Source: PLoS One. 2017 Aug 14;12(8):e0180736. doi: 10.1371/journal.pone.0180736 (PMC5555663; doi:10.1371/journal.pone.0180736)

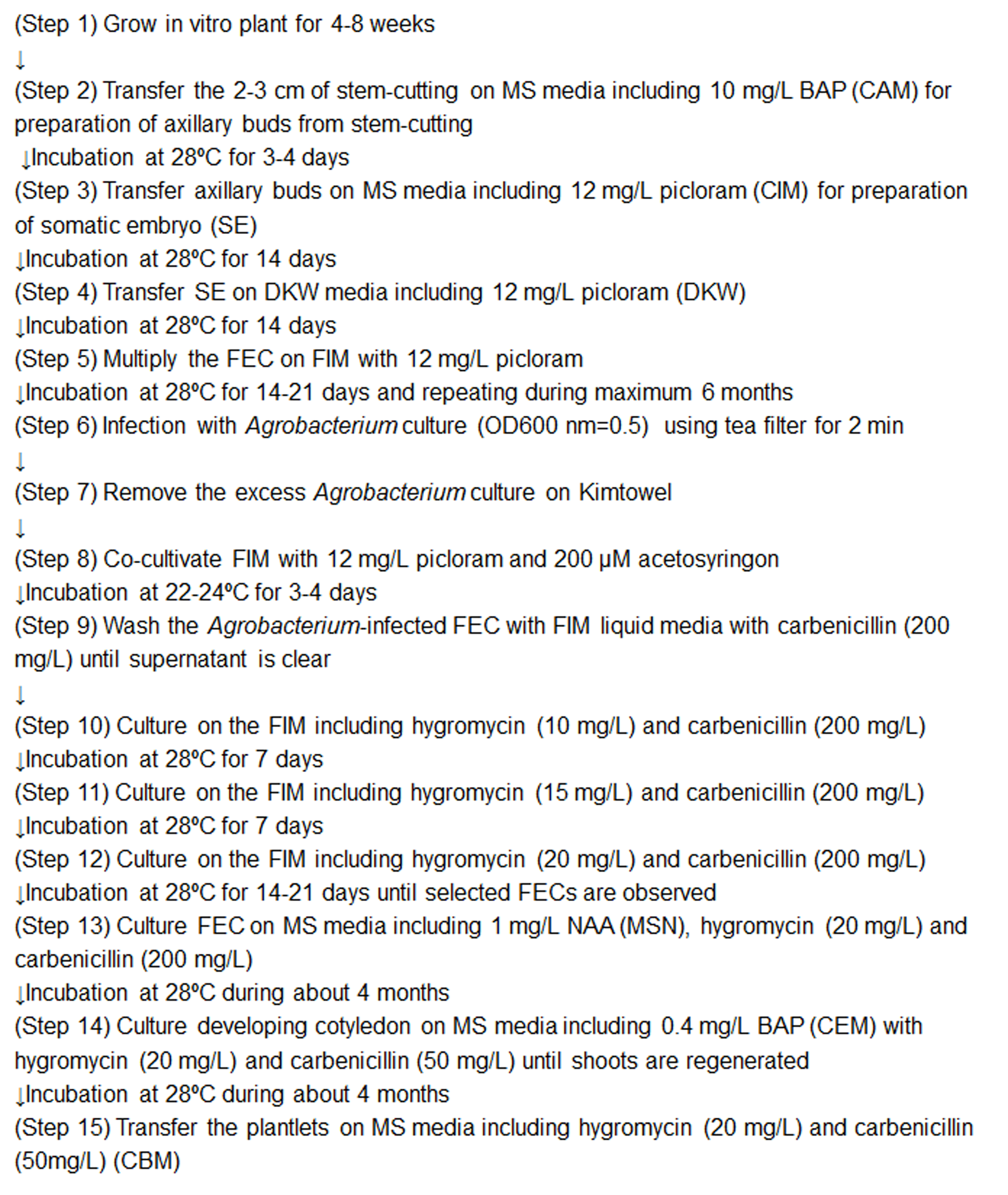

Supplement: S1 Fig — The present study was modified on the culture conditions in step 5. (TIF) [file pone.0180736.s001.tif]

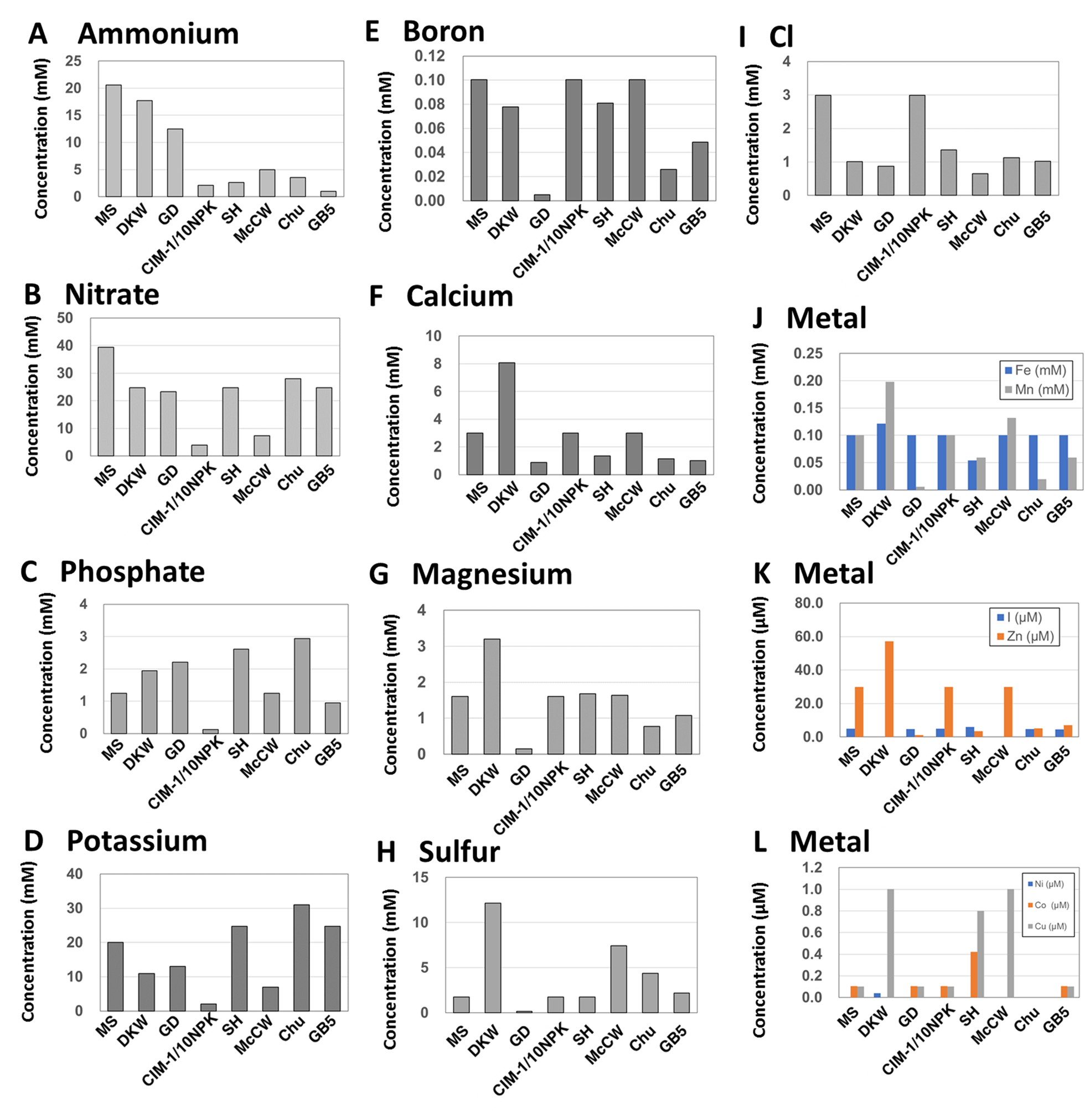

Supplement: S2 Fig — The concentration of ammonium (A), nitrogen (B), phosphate (C), potassium (D), boron (E), calcium (F), magnesium (G), sulfur (H), Cl (Cl), microelements (J), and other microelements (K and L) used in the various media. MS, DKW, GD SH, McCW, Chu and GB5 indicate Murashige and Skoog medium [47], Driver-Kuniyuki Walnut medium [48], Gresshoff and Doy medium [45], Schenk and Hildebrandt (SH) Basal Salt Mixture [49] McCown's Woody Plant Basal Salt Mixture [50], Chu (N6) Basal Salt Mixture [51] and Gamborg's B-5 Basal Salt Mixture [52], respectively. (TIF) [file pone.0180736.s002.tif]

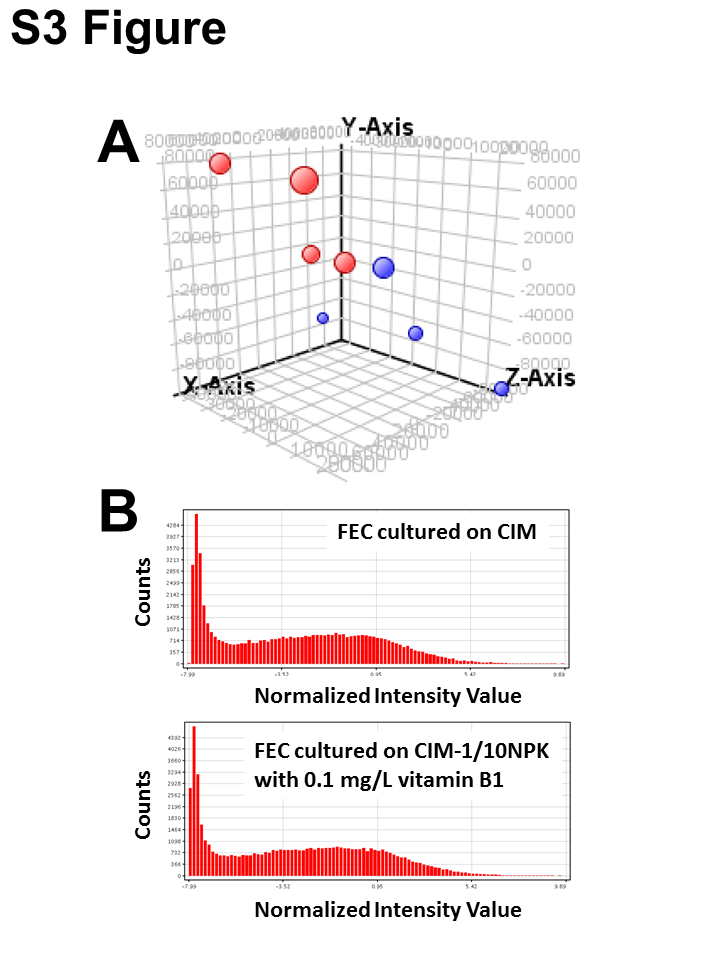

Supplement: S3 Fig — Principal component analysis of the gene expression identified by microarray (A), Frequency histogram of gene expression in microarray. The X-axis shows the normalized gene expression level. The Y-axis shows the number of genes at a given expression level (B). Red and blue circles on S2 Fig. A showed the FEC on CIM and the FEC on CIM-Ρ NPK, respectively. (TIF) [file pone.0180736.s003.TIF]

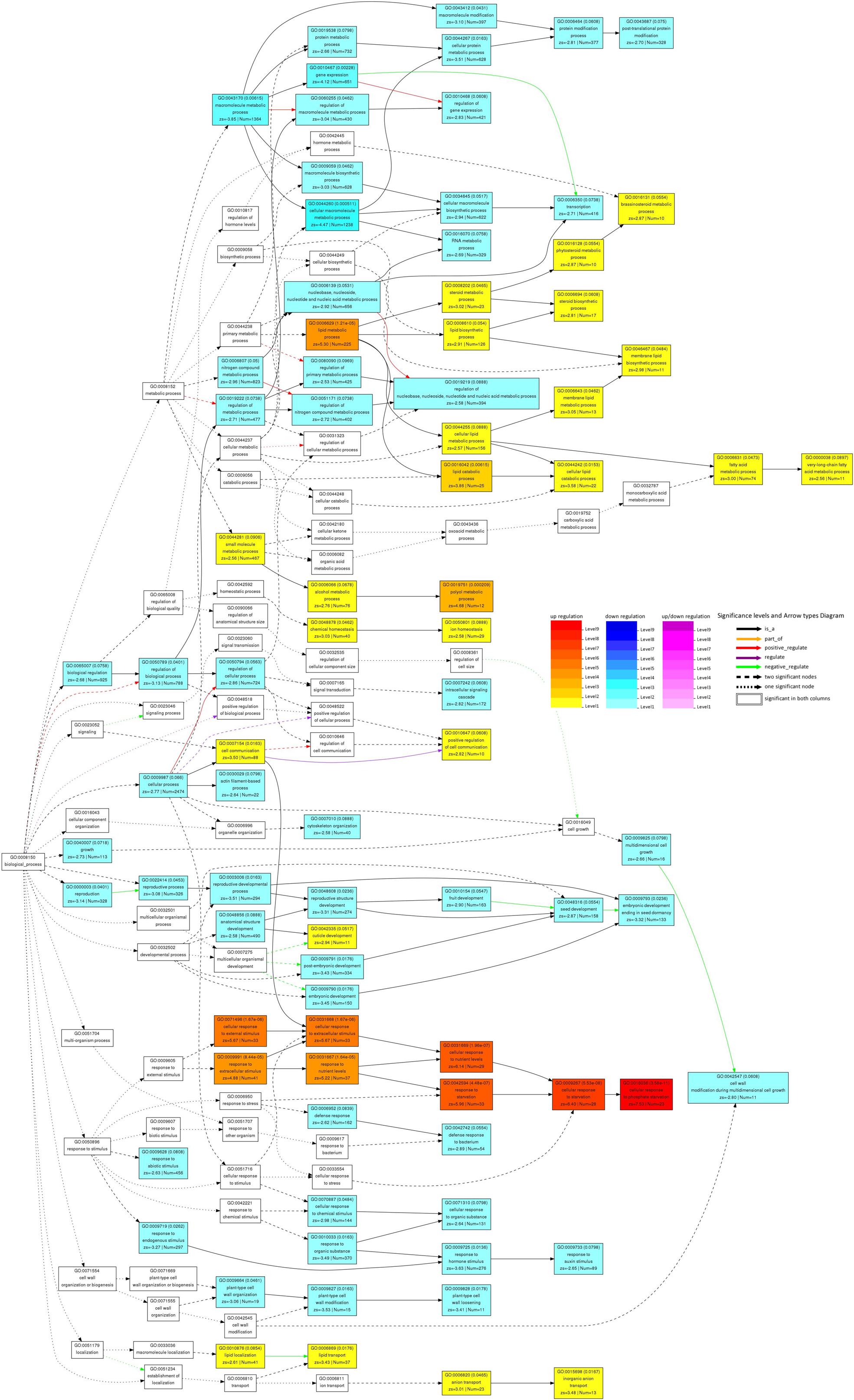

Supplement: S4 Fig — The graph was generated for the GO terms within the category biological process by Parametric Analysis of Gene Set Enrichment (PAGE) in agriGO [58]. A total of 10,959 genes, selected using the BH method (FDR<0.05), and annotated with an Arabidopsis gene code, were used in the analysis. The first pair of numbers represent the number of genes in the input list associated with the GO term and the number of genes in the input list, respectively. The second pair of numbers represent the number of genes associated with the GO term in the Arabidopsis database (TAIR9) and the total number of Arabidopsis genes with GO annotations in TAIR9. Box colors indicate the levels of statistical significance (FDR < 0.1 as determined using the Hochberg FDR). (TIF) [file pone.0180736.s004.tif]
